# Chronometry for the chorusing herd: Hamilton's legacy on context-dependent acoustic signalling—a comment on Herbers (2013)

**DOI:** 10.1098/rsbl.2013.1018

**Published:** 2014-01

**Authors:** Andrea Ravignani

**Affiliations:** 1 Department of Cognitive Biology, University of Vienna, Althanstrasse 14, Vienna 1090, Austria; 2 Language Evolution and Computation Research Unit, University of Edinburgh, Edinburgh, UK

**Keywords:** synchronization, chorusing, rhythm, selfish herd, origins of music, agent-based model

Biology Letters’ special feature on Hamilton's legacy pays due tribute to a brilliant mind. Herbers [1] and the other contributors paint a compelling picture of how Hamilton's work on inclusive fitness anticipated much contemporary evolutionary thinking, although sometimes not acknowledged until much later.

A more recent, although equally cited work by Hamilton is the ‘Geometry for the selfish herd’ [2], an elegant mathematical description of why individuals aggregate in space. In the spirit of this special feature [1], I illustrate why Hamilton's herd model should be recognized as an early mathematical formalism applicable to unrelated, although crucial, biological phenomena. Notably, Hamilton's model of gregarious behaviour can be directly applied to the problem of context-dependent acoustic signalling as follows, with the potential to describe how interdependent individual calls combine into choruses.

Many animals communicate acoustically, often with an emphasis on signal timing, rather than other acoustic properties [3]. Synchrony and chorusing occur in insects, amphibians, birds and mammals. An overarching question is how individuals ‘distribute’ their calls over time and why different individuals’ calls group together, leading to synchronous, alternating or phase-locked choruses [3]. Two hypotheses, suggested and tested in [4], predict clusters of calls: individuals could maximize overall sound intensity to attract females or, alternatively, individuals could call in quasi-synchrony to decrease the individual risk of predation. In both cases, individuals would tend to call close to each other, so to increase signal amplitude or alter individual conspicuousness (depending on the receiver), similarly to what happens in human applauding [5].

**Figure 1.**
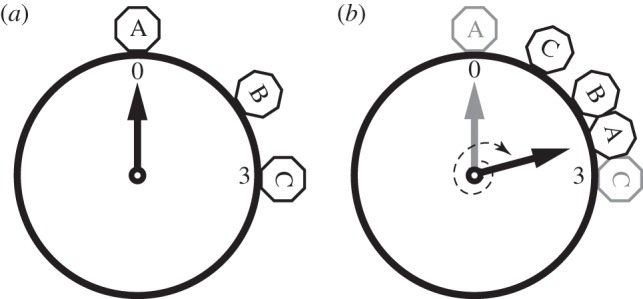
Clocks showing, for each individual, signalling time in two contiguous periods. Individual A signalled at 00.00, B at 02.00 and C at 03.00 (*a*). As agents choose when to call simultaneously, in the next time period (*b*), A remains silent for longer than a whole round (dashed thin arrow), postponing its call to 02.30. Similarly, C shifts its signal to 01.00. Adapted from [2].

Suppose three frogs, A, B and C, call periodically in time, say every second, although with different relative phases (see figure 1). B and C occur within a short time interval (short *silence*). A precedes them by a long interval (long *SILENCE*). The resulting acoustic pattern is A-*SILENCE*-B-*silence*-C-*SILENCE*-A- … A can modify its conspicuousness by shortening its ‘domain of silence’, i.e. timing its signal so it co-occurs, on average, with others’ calls. The most noise-robust, error-resistant strategy for A is to delay its call and signal exactly halfway between B and C; A calls, on average, in an ‘acoustically dense’ time period.

Assume, after Hamilton, that individuals A, B and C are located on a circular lily pond [2]. Instead of delaying or anticipating their signal phase/timing, they try to hide in-between other individuals. For instance, A occupies an isolated position on the pond, making it vulnerable to predators. A therefore seeks to decrease its ‘domain of danger’: if B and C are closer to each other than A is to any of them, A will jump and land between B and C [2].

Once formulated in these terms, it is clear how the mechanics of Hamilton's spatial predation model map one-to-one onto the acoustic signalling mechanism sketched here (table 1). The original spatial model featured a closed, circular space. Circular metaphors (e.g. clocks) are also appropriate to represent periodic events, and the ‘circular’ feature in [2] enables its direct application to periodic signals over time, as required in models of chorusing.

**Table 1.** Comparison between parameters from the one-dimensional model in [2] and the mathematically equivalent, context-dependent signalling framework sketched here.

gregarious behaviour [2]	context-dependent signalling (present model)
circular lily pond	time period (1 unit)
moving agent (e.g. frog)	acoustic signal produced by agent
agent location	time of signal produced by agent
movement clockwise or counter-clockwise	signal delay or anticipation
distance between two agents	time elapsed between two agents’ signals
‘domain of danger’	‘domain of silence’: the amount of silence, measured in time units, preceding and following a call

In both cases, a general model is derived from applying the basic ‘time shift’ mechanism to all individuals (cf. figure 1, A delays its call and C anticipates its), and dynamically over time (figure 1*a* versus *b*). At every time period, most individuals will have either changed location or adapted their calls, making previous decisions suboptimal and spurring individuals to compensate by jumping to a better location, or shifting the phase of their upcoming call to an acoustically denser period of time. Computer simulations for the predation model showed formation of clusters of individuals [2]. By analogy, group signalling dynamics should begin with randomly occurring individual calls scattered over time and converge towards a few, high-intensity acoustic peaks (produced by several near-synchronous individuals).

An additional, deeper mathematical link connects Hamilton's model of space with dynamical processes in time. Hamilton noted that only one initial configuration, three evenly spaced frogs, will prevent aggregation [2]; decades later, the mathematical investigation of rhythm and timing in biological systems found that the same initial configuration will prevent synchronization of oscillators in time [6].

Herbers admits that one volume cannot do full justice to Hamilton's genius, anticipating how his ideas will ‘influence the field over the coming 50 years’ [1]. Hopefully, as I show here, Hamilton's mathematical insights will inform future research on both rhythmic processes in humans, such as language and music, and context-dependent acoustic signalling in other species.
